# Prolonging exciton lifetime of WSe_2_ monolayer through image dipole interaction leading to huge enhancement of photocurrent

**DOI:** 10.1515/nanoph-2022-0590

**Published:** 2023-02-13

**Authors:** Kwang Jin Lee, Jae-Pil So, Sandeep Kumar Chamoli, Hoo-Cheol Lee, Hong-Gyu Park, Minhaeng Cho

**Affiliations:** Center for Molecular Spectroscopy and Dynamics, Institute for Basic Science (IBS), Seoul 02841, Republic of Korea; Department of Physics, Korea University, Seoul 02841, Republic of Korea; GPL, Photonics Laboratory, CIOMP, Changchun, China; Department of Chemistry, Korea University, Seoul 02841, Republic of Korea

**Keywords:** 2D transition-metal dichalcogenides, exciton, image dipole, metamaterials

## Abstract

Two-dimensional transition metal dichalcogenides (2D TMDs) have been demonstrated as one of the most outstanding materials not only for fundamental science but also for a wide range of photonic applications. However, an efficient way to control their excitonic properties is still needed for advanced applications with superior device performance. Here, we show that the exciton lifetime of WSe_2_ monolayer can be prolonged using metamaterials. We observe a ∼100% reduction in the electron-hole recombination rate of WSe_2_ monolayer placed on a hyperbolic metamaterial substrate and demonstrate that such a remarkable change results from the destructive image dipole interaction with the in-plane exciton transition dipole. Furthermore, this substantial increase in exciton lifetime leads to order-of-magnitude (10-fold) enhancement of photocurrent in the 2D WSe_2_-based hybrid photodetector with metamaterials. Tailoring the optical transition properties of 2D TMD materials with specially designed metamaterials, demonstrated here, will pave the way for developing 2D material-based optoelectronics.

## 1 Introduction

Excitons, electron-hole pairs created by a photoexcitation, generally dominate the optical properties of semiconductors and are highly relevant for a variety of optoelectronic applications, including solar cells, photodetectors, and light-emitting devices. Once generated, they undergo relaxation via various decay processes depending on the material’s intrinsic band structure as well as external conditions such as excitation power and local environments. Hence, tailoring exciton lifetime has been a formidable task in bulk semiconductors owing to numerous additional effects. However, efficient control of the recombination process of excitons has become rather straightforward with the emergence of two-dimensional (2D) semiconductors, most notably transition metal dichalcogenides (TMDs) [1].

The family of 2D TMDs is a promising material platform not only for the fundamental nature of 2D systems but also for potential applications in a wide range of optoelectronics and nanophotonics [2–5]. In addition, these materials have provided new opportunities to engineer light–matter interactions, including single-photon emitters [6–8] and polaritonic applications [9–13]. The recent advances in optoelectronics associated with these materials are based on strong exciton binding energy and notable photoluminescence in the visible to near-IR spectral range. The 2D nature of monolayer TMDs enhances the Coulomb interaction, resulting in strongly bounded excitons that can dominate the optical and charge-transport properties [14–19]. Therefore, a thorough understanding of the relationship between exciton and its electrostatic environment is critical for revealing the underlying physics related to exciton relaxation in such materials, which can lead to the development of innovative nanophotonic devices.

The orientation of radiative excitons is also one of the most crucial parameters in designing the integrated photonic applications requiring highly directional light since it depends on their electronic properties and dipole selection rules [20–24]. Although most semiconducting materials have shown mixed dipole orientations, various low-dimensional semiconductor structures have recently been found to be either in-plane or out-of-plane orientation. Typically, 2D semiconductors possessing bright intra-layer excitons with an in-plane orientation of transition dipole favour directional out-coupling of radiation. The preferential orientation of strained TMDs have recently been studied by using nanophotonic structures such as nanogap plasmons [25] and micropillars [26]. In particular, it has been demonstrated that strain-induced quantum emitters in WSe_2_ undergo a transition from in-plane to out-of-plane dipole moment orientation and show exhibit photon emission properties [26]. While numerous previous studies have focused on the excitonic properties of 2D TMDs, direct utilization of their preferential orientation for optoelectronic applications is still lacking.

## 2 Methods

In this article, we show that the excitonic properties of a well-characterized WSe_2_ monolayer can be modulated by introducing metamaterial substrates. The interaction between in-plane transition dipole in WSe_2_ monolayer and its image formed by the substrate metamaterial significantly reduces the recombination rate. Numerical simulations of the Purcell factor depending on the dipole orientation with metamaterials have been systematically performed to examine the role of image dipole interactions. Notably, the exciton recombination time in the WSe_2_ monolayer becomes significantly longer in the presence of metamaterials. Estimation of quantum yield indicates that the image dipole interactions are predominant over the quenching effect of the emission in the near field regime, which results in a gigantic enhancement of photocurrent in the photodetector devices in the presence of metamaterials close to the 2D WSe_2_ monolayer.

**Figure 1:**
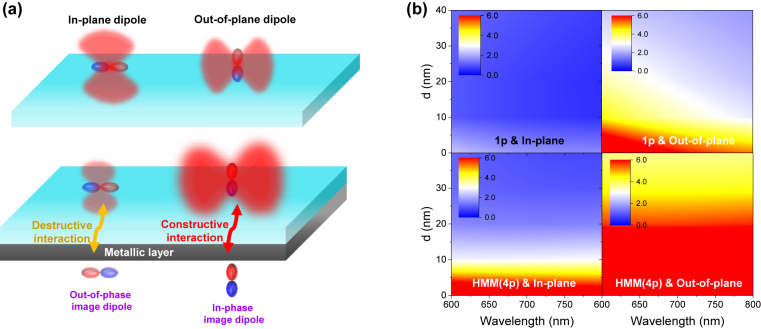
Schematic description of image dipole interaction and calculation of Purcell factor depending on the dipole orientations. (a) Schematics of in-plane and out-of-plane transition dipoles without (top) and with (bottom) HMM substrate, in which in-plane (out-of-plane) dipole moment decreases (increases) due to destructive (constructive) interaction with its image dipole. (b) Numerical simulations of Purcell factor for in-plane and out-of-plane dipoles, as functions of *d* and wavelength in the presence of 1p and HMM (4p) for the substrate.

Figure 1a shows how the in-plane and out-of-plane transition dipoles can be affected by metallic substrate. The relevance of dipole orientation can be seen by considering the metallic layer that produces the corresponding image dipoles. Based on the in-plane transition dipole characteristics of the WSe_2_ monolayer demonstrated in the previous studies [22, 24, 27], we describe the effect of an image dipole on the transition dipole moment where the in-plane image dipole cancels out the real dipole moment. On the other hand, the out-of-plane transition dipole constructively interacts with the in-phase image dipole, resulting in an enhanced dipole moment. Blue and red colors indicate electron and hole wavefunctions, respectively, and the strength of the transition dipole moment is represented by the depth of color. The relatively lighter color of an in-plane dipole (bottom left in Figure 1a) in the presence of a metallic substrate than that in a glass substrate indicates the weaker strength of the net transition dipole moment resulting from the destructive interaction with an out-of-phase image dipole. On the other hand, the relatively denser color of an out-of-plane dipole indicates the stronger strength of the net transition dipole moment resulting from the constructive interaction with an in-phase image dipole [28]. This variation of dipole strength is consistent with the spatial electric field distributions for a single dipole and two dipoles with in-plane and out-of-plane orientations (Figure S1).

## 3 Results

We considered various metamaterials, including metallic layers, to modulate such excitonic dipoles of 2D TMDs. In particular, we focused on hyperbolic metamaterials (HMMs) that have been extensively studied over the past years due to their unique optical properties originating from the high-*k* states [29–31]. Furthermore, they have been shown to exert nonlocal effects on the various photophysical properties such as inter- and intramolecular charge transfers and electron tunneling by altering the dielectric environment [32–35], which suggests that one can control the optical properties of optoelectronic materials without complicated molecular engineering. Since HMMs have been considered helpful for tuning radiative and nonradiative properties of semiconducting materials, we used HMMs to control the excitonic properties of 2D TMDs.

To quantitatively describe the image dipole effect on transition dipoles, we calculated the Purcell factor (PF), which defined as the ratio of spontaneous emission in a cavity or cavity-like structure to that in free space, for the in-plane and out-of-plane dipoles placed at a distance *d* from the 1p and HMM substrates as a function of wavelength (*λ*) (Figure 1b). Here, single pair and four pairs of 10 nm thick Ag and Al_2_O_3_ layers are referred to as 1p and HMM, respectively. The PF is calculated as the ratio of the power of luminescence in the far-field to that emitted by the dipole in an infinite uniform medium. For an in-plane dipole on 1p, interestingly, PF < 1 is possible for *λ* ≳ 650 nm and a specific range of *d*. On the other hand, the PF is always larger than 1 for an out-of-plane dipole, meaning that the out-of-plane dipole boosts the PF at short distances, i.e., the constructive interference between the out-of-plane and its image increases the PF in addition to the excitation of surface waves on the metallic substrates. We also note that there exists a spectral and spatial range where the PF < 1 even on an HMM for an in-plane dipole even though the spectral and spatial range where PF > 1 is wider than the case of 1p substrate owing to the high-*k* modes supported by HMMs.

**Figure 2:**
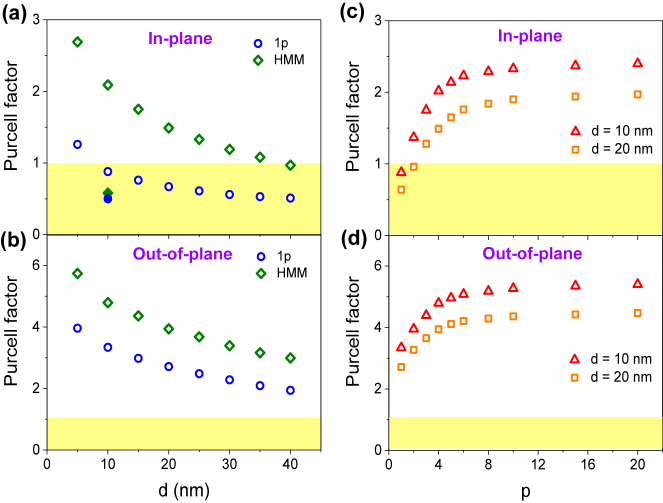
Dipole orientation dependent Purcell factor in the presence of metamaterials. (a, b) Calculated Purcell factors at a wavelength of 750 nm as a function of *d* in the presence of 1p and HMM substrates for in-plane (a) and out-of-plane dipoles (b), respectively. Filled dots in (a) show experimental results. (c, d) Calculated Purcell factors as a function of *p* at *d* = 10 and 20 nm for in-plane (c) and out-of-plane dipoles (d), respectively. The yellow region indicates that the Purcell factor is less than 1.

**Figure 3:**
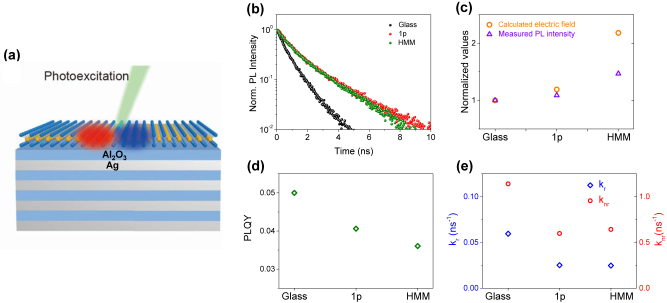
Change in photophysical properties due to 2D nature of WSe_2_ monolayer. (a) Schematic illustration of experimental configuration exhibiting a WSe_2_ monolayer on metamaterial substrate consisting of Ag and Al_2_O_3_ alternative layers. Blue and red colors represent electron and hole wavefunctions, respectively. (b) Measured time-resolved photoluminescence of WSe_2_ monolayers on glass (black), 1p (red), and HMM (green) substrates. (c) Plots of calculated electric-field intensity (orange) and measured PL intensity of WSe_2_ monolayers (purple) on glass, 1p, and HMM substrates. The electric field intensity and PL intensity are normalized by the corresponding values of the glass substrate. (d) Estimation of photoluminescence quantum yield WSe_2_ monolayers on glass, 1p, and HMM substrates. (e) *k*_*r*_ and *k*_*nr*_ values for glass, 1p, and, HMM substrates.

To show the dependence of the PF on the structural parameters of metamaterial substrates, we plot PF as a function of *d* and the number of Ag–Al_2_O_3_ pairs, *p* (Figure 2). Figure 2a and b displays the *d*-dependence of the PF at *λ* = 750 nm for in-plane and out-of-plane dipoles, respectively, which corresponds to the cross-section of Figure 1b at *λ* = 750 nm. The filled dots shown in Figure 2a indicate the experimental results to be shown in Figure 3a, which we will discuss later. For both dipole orientations, the calculated Purcell factors decrease with *d*, which is mainly due to the reduction of near-field couplings associated with surface plasmon polaritons (SPPs) and bulk plasmon polaritons. As can be seen in Figure 2a, the Purcell enhancement (PF > 1) is pronounced even for in-plane dipoles up to *d* = 10 nm and 30 nm in the presence of 1p and HMM, respectively. We also note that the optical field enhancement due to SPPs could contribute to the Purcell enhancement. More interestingly, Figure 2a clearly shows the PF less than one as *d* becomes larger than 10 nm, which can be solely attributed to the destructive interaction with the in-plane image dipole. In Figure 2c and d, we examine the limiting behavior of PF upon increasing *p*. Not only do we confirm the saturating behavior, but we also note that it is hard to see the PF < 1 at small *d* when the number of metal-dielectric pairs is sufficiently large, which can be attributed to the contribution of coupling with volume plasmon polaritons that increases decay rate [30, 36, 37] (see Figure S2).

The schematic illustration in Figure 3a shows the experimental configuration under investigation. Excitons generated upon photoexcitation in a WSe_2_ monolayer placed on a metamaterial substrate consisting of Ag and Al_2_O_3_ thin alternating layers can be affected by their images. Four pairs of Ag and Al_2_O_3_ (4p) layers were used as a hyperbolic metamaterial (HMM), fabricated by electron beam evaporation. The thickness of every single layer is 10 nm, and its effective dielectric permittivity is shown in Figure S3 (Supplementary Material) exhibiting hyperbolic dispersion throughout the visible frequency range. The top layer of 1p and HMM is always a 10 nm thick Al_2_O_3_ layer so that the direct contact between an emitter and the metal layer can be avoided. We also prepared a single pair of Ag and Al_2_O_3_ (1p) substrates for reference. Monolayer WSe_2_ was mechanically exfoliated from single-crystalline bulk WSe_2_ (HQ Graphene) and identified by optical characterization. Then, the WSe_2_ monolayer flakes were transferred onto the metamaterial substrates using a PDMS stamping method.

We then measured the time-resolved photoluminescence (TRPL) of WSe_2_ monolayers (Figure 3b). The samples were optically pumped by a supercontinuum pulsed laser (NKT Photonics) with a repetition rate of 20 MHz and an excitation power of 40 μW at 532 nm. The diameter of the beam spot is 2 μm. Then, the photon statistics was measured by time tagging electronics (PicoQuant Picoharp 300). The measured decay time constants for all three substrates were analyzed by bi-exponential fitting and summarized in Table S1 (Supplementary Material). Observing two different time components indicates that exciton recombination is strongly affected by nonradiative decay mainly due to trap (or defect) states induced by W and Se vacancies [38]. We note that it is hard to exactly separate out radiative component from fitting results since radiative recombination takes place throughout entire time scale (see Supplementary Material), naturally lead us to consider the average lifetimes (denoted as $\bar{\tau }$ ) obtained by considering the sum of normalized amplitude-weighted decay times to discuss the image dipole interaction.

We found that $\bar{\tau }$ of WSe_2_ monolayer deposited on glass, 1p, and HMM are ${\bar{\tau }}_{\text{glass}}$ = 0.83 ns, ${\bar{\tau }}_{1p}$ = 1.62 ns, and ${\bar{\tau }}_{\text{HMM}}$ = 1.49 ns, respectively, yielding ${\bar{\tau }}_{\text{glass}}/{\bar{\tau }}_{1p}$ = 0.51, and ${\bar{\tau }}_{\text{glass}}/{\bar{\tau }}_{\text{HMM}}$ = 0.56, respectively. The slightly shorter ${\bar{\tau }}_{\text{HMM}}$ compared to ${\bar{\tau }}_{1p}$ is due to the higher photonic density of states and more active nonradiative coupling with volume plasmon polaritons in HMM [30, 36, 37]. In addition, such a small difference in the exciton lifetimes of WSe_2_ monolayers on 1p and HMM substrates can be explained by the fact that the strength of image dipole interaction decreases with increasing the distance between the real dipole and its image. Therefore, the effects of image dipoles far from the WSe_2_ monolayer could be weak so that the extent of prolonged exciton lifetime does not linearly depend on the number of metal-dielectric pairs.

These low ratio of recombination time indicate that the exciton lifetime of the WSe_2_ monolayer can be significantly prolonged by the 1p and HMM substrates, which seems to be counterintuitive given the fact that an HMM is a well-known metamaterial that can enhance the Purcell effect due to high photonic density of states [39–41]. Our observation is also contrary to a quenching effect resulting from the near field coupling based on direct coupling between dipole field and SPPs. Since the SPPs are nonradiative modes generated at the metal-dielectric interface, coupling with SPPs usually reduces the PL lifetime of an emitter by increasing the local density of state. In the case of conventional emitters of which transition dipoles are randomly oriented, the dipole orientation does not play a role in determining the PL lifetime (exciton lifetime). In the current study, however, strong anisotropy of dipole orientation originating from the 2D nature of the WSe_2_ monolayer exhibits an unusual increase in the PL lifetime even for sufficiently small *d* of 10 nm. In addition, this way of prolonging the exciton lifetime by reducing the net transition dipole moment due to an in-plane orientation radically differs from the conventional approach using photonic nanocavities to realize low photonic density of states [42]. Consequently, experimental results shown in Figure 3b lead us to conclude that the destructive interaction between the in-plane transition dipole and its image formed by a metallic layer is predominant over the quenching effect by the underlying metallic layers.

We also measured the exciton lifetime of a WSe_2_ monolayer deposited on HMM with a top layer of 30 nm-thick Al_2_O_3_ (Figure S4), exhibiting a slightly longer exciton lifetime than in the HMM substrate. This result is qualitatively consistent with numerical calculations that show a lower Purcell factor with increasing *d* (Figure 2a). As aforementioned, experimental data points obtained from Figure 3b are marked in Figure 2b, which are different from the numerical simulation. We attribute this discrepancy to the fact that the WSe_2_ monolayer exhibits an extremely low quantum efficiency of less than 5% due to substantial nonradiative processes originated from defects, vacancies, and carrier density [43, 44]. In addition to low quantum efficiency, small size of WSe_2_ monolayer flake hinders direct measuring photoluminescence quantum yield (PLQY) using integrating sphere [45]. Since direct measurement of the absolute PLQY is not applicable for 2D TMDs, the alternative way to measure the PLQY using reference samples such as thin films of uniformly dispersed fluorescent organic dyes has been used [43]. However, the preparing such a homogenous film is still technically challenging.

## 4 Discussion

In this study, we note that the relative variation of PLQY depending on the substrates can be estimated by theoretical analysis combining with PL lifetime. Thus we examine the behavior of radiative and nonradiative decay processes separately, enabling us to understand the discrepancy between experimental results and numerical simulation. The definition of PLQY (Φ) is given by,

(1) $$\Phi =\frac{\#\;of\;\;\;emitted\;\;\;photons}{\#\;of\;\;\;absorbed\;\;\;photons}\equiv \frac{P}{A}.$$

Here, we define Φ depending on the substrates as Φ_glass_ ≡ *P*_glass_/*A*_glass_, Φ_1*p*_ ≡ *P*_1*p*_/*A*_1*p*_, Φ_HMM_ ≡ *P*_HMM_/*A*_HMM_. For each case, the numerator (the number of emitted photons) is proportional to the PL intensity shown in Figure S5 (Supplementary Material), where the normalized steady-state PL spectra of WSe_2_ monolayers on glass, 1p, and HMM substrates are plotted. The spectral shape, bandwidth, and peak position are independent of substrates, indicating that no additional effect is expected to occur (Figure S5c shows reproducibility of TRPL measurement). In Figure 3c, we plot the PL intensities, assuming that the PL intensity of the WSe_2_ monolayer on a glass substrate (violet triangle) is unity. Higher PL intensities of WSe_2_ monolayers on metallic substrates are due to the increased absorption or the enhanced local electric field at the optical frequency [46]. To estimate the number of absorbed photons, we take into account the electric field intensity at the emitter’s position. We note that the absorbance (*A*) proportional to the number of absorbed photons is the function of electric field intensity $I\left(t\right)$ , given by $\frac{1}{2c\mu_{0}}{\left|E\left(t\right)\right|}^{2}$ , where *E*(*t*) is the electric field generated by incident light at time *t*. To obtain the electric field intensity at the position of WSe_2_ monolayer, we numerically calculate the static electric field intensity profiles for the three different substrates (Figure S6 in Supplementary Material). We estimate the ratio of electric field intensity at the position of WSe_2_ monolayer for 1p and HMM to that for glass to be *A*_1*p*_ = 1.18*A*_glass_ and *A*_HMM_ = 2.23*A*_glass_, respectively, which are also plotted in Figure 3c (orange circle). From the comparison between the calculated electric field intensities and the experimentally measured PL intensities, we expect that the quantum yields of WSe_2_ monolayers on 1p and HMM substrates are smaller than that on glass, since the increment of PL intensity due to the presence of 1p and HMM is smaller than that of absorption.

Based on the previous studies reporting that Φ of WSe_2_ monolayer on glass is estimated to be 0.05 for a wide range of excitation power [44, 45], we can obtain Φ for each substrate by substituting the number of emitted photons with normalized PL peak intensity and the number of absorbed photons with the normalized electric field intensity. 18% and 29% decreases of Φ for 1p and HMM substrates, as shown in Figure 3d, respectively.

In general, a decrease in PLQY of conventional bulk emitters near metallic substrate is understandable since PL quenching due to near field coupling usually reduces the number of emitted photons [47]. Thus, we need an in-depth analysis to elucidate the characteristic difference between bulk and 2D emitters. To examine the radiative and nonradiative decay processes further, let us consider another representation of Φ,

(2) $$\Phi =k_{r}\tau$$

where *k*_*r*_ is the radiative decay rate. The PL lifetime *τ* is given by 1/(*k*_*r*_ + *k*_*nr*_) where *k*_*nr*_ is the nonradiative decay rate. Using Eq. (2), we could evaluate the variations of both *k*_*r*_ and *k*_*nr*_ using the experimentally measured Φ and *τ* values for different substrates. As shown in Figure 3e, 18% (29%) decrease in Φ and 96% (79%) increase in τ for a 1p (HMM) substrate result in 59% (61%) decrease in *k*_*r*_. Based on these values, we estimate 46% and 45% decreases in *k*_*nr*_, for 1p and HMM, respectively (see Supplementary Information).

In the case of conventional emitters on a metallic layer, an increase in *k*_*nr*_ is accompanied by a reduction of *k*_*r*_, the increase in *k*_*nr*_ usually exceeds the reduction of *k*_*r*_, which leads to the decreases in both *τ* and Φ. In the case of 2D WSe_2_, however, we find that both *k*_*r*_ and *k*_*nr*_ significantly decrease in the presence of metamaterials as shown in Figure 3e. Here, we found evidence on the role of HMMs in the reduction of *k*_*r*_, which increases the exciton lifetime even though the contribution from *k*_*r*_ to the overall lifetime is smaller than that from *k*_*nr*_. Furthermore, it is shown that a considerable reduction of *k*_*nr*_ due to the presence of HMMs results in an increase in the exciton lifetime. Such a decrease in *k*_*nr*_ due to metallic substrates is counterintuitive since the near field coupling actively occurs irrespective of dipole orientation. In addition, decrease in *k*_*nr*_ is hard to be understood since the image dipole interaction seems to be associated with transition dipole moment determining the radiative decay only. However, it should be noted that recent studies reported that various nonradiative processes, including charge transfer, exciton annihilation, energy transfer, and Auger recombination, are strongly affected by nanocomposite structures [32, 35, 48, 49]. Thus, it is believed that *k*_*nr*_ can be indirectly influenced by image dipole interaction. For example, it was shown that the image dipole interactions can suppress the biexciton Auger recombination in colloidal quantum dot films on metamaterials, where the transition dipole of each quantum dot could be assumed to be isotropic [49]. This implies that the image dipole interaction based on strong dipole anisotropy exhibited by 2D TMDs can further suppress the Auger process, likely leading to a decrease in *k*_*nr*_. Another factor needed to be considered is the excitation trapping process occurring between conduction band edge and trap states formed by defects or vacancies [38, 50]. Although the trapping process is not directly associated with the variation of transition dipole moment (see Table S1), its contribution to the recombination process could not be included in our numerical simulations. Therefore, given that pure transition dipole (100% radiative decay) is assumed to be nearby metallic substrates in the numerical calculations shown in Figure 1b, the ratio ${\bar{\tau }}_{\text{glass}}/{\bar{\tau }}_{1p}$ and ${\bar{\tau }}_{\text{glass}}/{\bar{\tau }}_{\text{HMM}}$ should not be the same as the PF (Figure 2). In other words, the discrepancy between the numerical simulations and experimental results occurs because various nonradiative processes including trapping process cannot be fully considered in the simulations.

**Figure 4:**
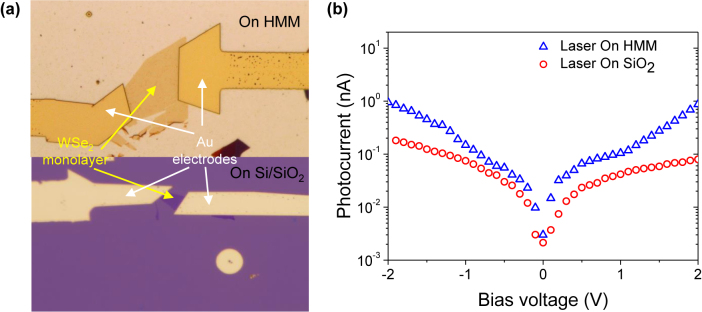
Enhancement of the photocurrent resulting from prolonged exciton lifetime. (a) Optical microscope images of WSe_2_ photodetectors on SiO_2_ (bottom) and hyperbolic metamaterial (top). (b) Measured photocurrent of the devices in (a); WSe_2_ monolayer on HMM (blue) and SiO_2_ (red). Bias voltage varies while the 532 nm laser is incident.

Finally, the longer exciton lifetime achieved by dipole-image dipole interaction in the in-plane direction can lead to considerably higher photocurrent [51]. This feature will be useful for high-performance optoelectronic applications such as a photodetector producing remarkably high photocurrent. To realize this idea, we fabricated a hybrid photodetector with an HMM substrate. Figure 4a shows the optical microscope images of the photodetectors with WSe_2_ monolayer on HMM (top) and Si/SiO_2_ substrates (bottom). The latter was considered for the sake of comparison. In order to fabricate these devices, the mechanically exfoliated WSe_2_ monolayer flakes were transferred onto the substrates, and then ∼5 nm of Ti and 100 nm of Au electrodes were deposited using a thermal evaporator. The schematics of the photodetector configuration are presented in Figure S7 (Supplementary Material). We note that our photodetector devices are composed of monolayer semiconductors without band structure engineering, but with rationally designed substrates capable of increasing the photocurrent response only by optical effects. The photocurrent measurement was performed by applying a bias voltage to the devices using a source measurement unit while they were illuminated by a 532 nm continuous-wave solid-state laser used in TRPL measurement. As shown in Figure 4b, we obtained approximately 10-fold higher photocurrent at a bias voltage of ±2 V by introducing the HMM substrate (Figure S8 shows reproducibility of photocurrent measurement).

To clarify that image dipole interaction is mainly responsible for this considerable enhancement, we discuss the photophysical parameters associated with photocurrent. We note that three parameters, the number of generated excitons (*N*_*G*_), exciton transport property (*η*_*D*_), and free carrier mobility (*μ*), play critical roles in determining the photocurrent (*i*_photo_) [51], i.e., *i*_photo_ ∝ *N*_*G*_⋅*η*_*D*_⋅*μ*.

Regarding the number of generated excitons (*N*_*G*_), one might infer that the enhancement of photocurrent dominantly due to an increase of absorbed photons caused by the stronger field intensity formed by metamaterials. However, the reduction in PLQY in Figure 3d and SI (Supplementary Note I) clearly shows that the number of available radiative excitons to produce photocurrent rather decreases in the presence of metamaterial substrates. Even if we take into account the contribution of absorption enhancement due to metamaterials, it is still insufficient to explain the order-of-magnitude increase in photocurrent. Next, exciton transport property (*η*_*D*_) is strongly related to exciton diffusion length, which is the maximum length an exaction can migrate before germinate recombination occurs. Longer exciton diffusion length means more chances of reaching the electrodes, which eventually leads to a higher photocurrent. It is worth noting that exciton diffusion length (*L*_*D*_) is determined by exciton lifetime using the relationship given by $L_{D}\propto \sqrt{D\tau }$ , where *D* and *τ* are the diffusion coefficient and exciton lifetime, respectively [52, 53]. Since the diffusion coefficient is predominantly dependent on exciton density fluctuation and is not likely affected by metamaterials [48, 54, 55], longer exciton lifetime (shown in Figure 3b) due to image dipole interactions leads to longer diffusion length, which consequently results in higher photocurrent. Finally, it can be assumed that the charge mobility (*μ*) of WSe_2_ monolayer is hardly affected by metamaterials because it is an intrinsic property of active materials.

We also note that the PLQY of an emitter is not directly related to device performance. In the case of a solar cell, for example, the PLQY of active material is not the same as the external quantum efficiency (EQE) that is the ratio of the number of charge carriers collected by the solar cell to the number of photons of a given energy shining from outside. We also consider many-body interaction such as exciton-exciton annihilation that actively takes place in 2D TMDs. We note that those phenomena resulting from higher electric field caused by metamaterials (Figure S6) can be detrimental for photovoltaics and photodetectors since they generally give rise to shorter exciton lifetime. However, the order-of-magnitude higher photocurrent obtained in the hybrid photodetector enables us to rule out those additional effects. Therefore, the above discussions lead us to conclude that the reduction of the net transition dipole moment due to an image dipole interaction with in-plane orientation can become another underlying mechanism to enhance the device’s efficiency.

## 5 Conclusion

In conclusion, we observe the substantial increase in the exciton lifetime in the WSe_2_ monolayer by introducing metamaterial structures, which is attributed to the image dipole interaction occurring between in-plane transition dipoles and their images. The estimation of quantum yield provides that both radiative and nonradiative decay rates significantly decrease in the presence of an HMM, consistent with the reduction of the net transition dipole moment resulting from the destructive image dipole interaction. This study is also somewhat consistent with the previous study that a partially reflecting mirror can be used to enhance the lifetime of polaritons in 2D materials dramatically [56]. Furthermore, we developed the hybrid photodetector that exhibits an order-of-magnitude higher photocurrent due to the prolonged exciton lifetime through image dipole interactions. This approach will be a valuable strategy to prolong the exciton lifetime that predominantly determines the device performance of optoelectronic applications.

## Supplementary Material

Supplementary Material Details
