# Supplementary material for: Prolonging exciton lifetime of WSe2 monolayer through image dipole interaction leading to huge enhancement of photocurrent
Source: Nanophotonics. 2023 Feb 13;12(4):695–703. doi: 10.1515/nanoph-2022-0590 (PMC11636519; doi:10.1515/nanoph-2022-0590)
Supplement: Supplementary file 1 — Supplementary Material Details [file j_nanoph-2022-0590_suppl_001.docx]

**Prolonging exciton lifetime of WSe_2_ monolayer through image dipole interaction leading to huge enhancement of photocurrent**

**Kwang Jin Lee^1†^, Jae-Pil So^2†^, Sandeep Kumar Chamoli^3^, Hoo-Cheol Lee^2^, Hong-Gyu Park ^1,2^*, and Minhaeng Cho^1,4^***

^1^ Center for Molecular Spectroscopy and Dynamics, Institute for Basic Science (IBS), Seoul 02841, Republic of Korea.

^2^ Department of Physics, Korea University, Seoul 02841, Republic of Korea

^3^ GPL, photonics Laboratory, CIOMP, China

^4^ Department of Chemistry, Korea University, Seoul 02841, Republic of Korea.

*e-mail: [mcho@korea.ac.kr](mailto:mcho@korea.ac.kr), hgpark@korea.ac.kr

†These authors contributed equally.


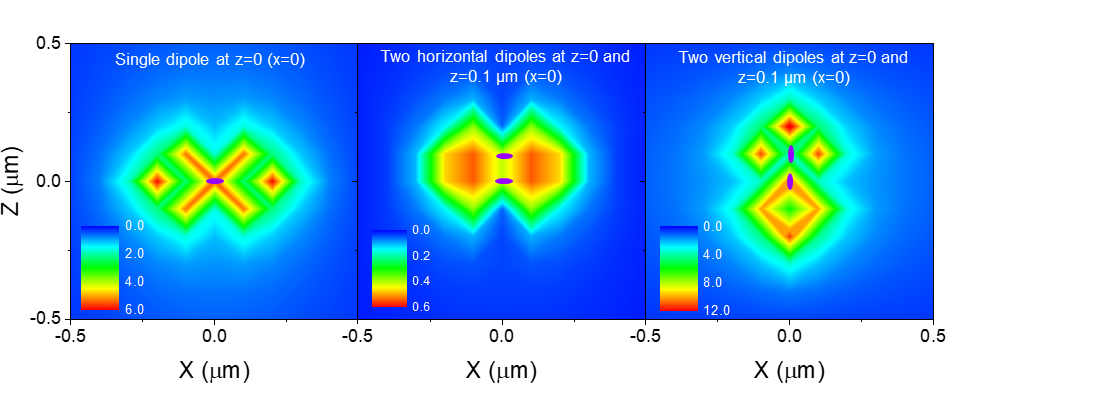


**Figure S1.** Spatial distributions of electric field intensities around a single dipole, two horizontally oriented dipoles, and two vertically oriented dipoles calculated by using a FDTD method. The positions of dipoles are marked by violet ellipses.

**Figure S2**. Reflectance spectra of 1p and 4p (HMM) substrates. The reflection dip at ~ 330 nm shown for a 1p substrate is due to surface plasmon resonance of silver film. For HMM (4p), this dip still can be seen and corresponds to the volume (bulk) plasmon polaritons (VPPs) originating from coupling of surface plasmon polaritons in the metal-dielectric multilayer super lattice.


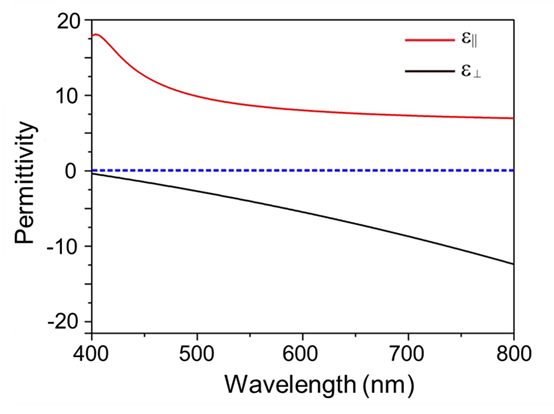


**Figure S3.** Effective dielectric functions along with in-plane (ε_∥_) and out-of-plane (ε_⊥_) directions, calculated by effective medium theory. Type II hyperbolic dispersion can be seen throughout the visible wavelength region.


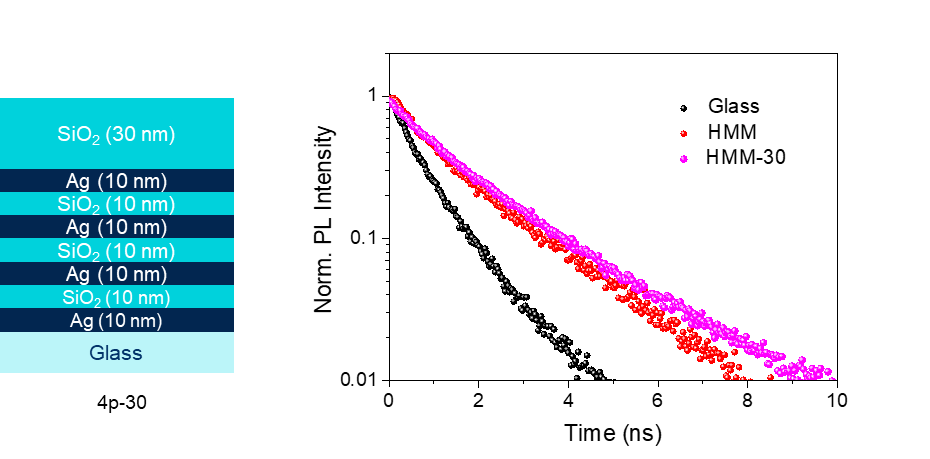


**Figure S4.** HMM with 30 nm-thick Al_2_O_3_ top layer (left) and the measured PL lifetime in the substrates with glass and HMM (right).


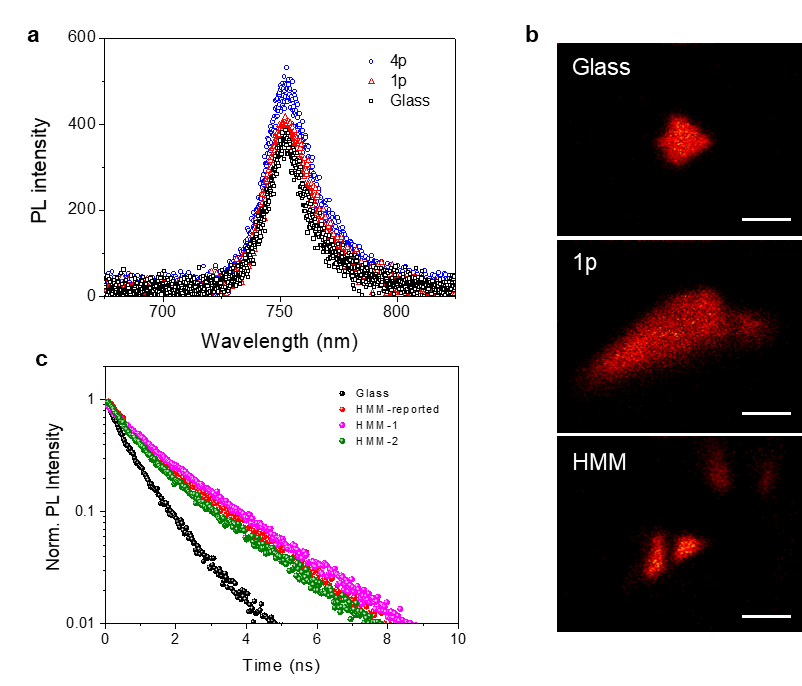


**Figure S5.** **a,** Steady-state photoluminescence spectra of WSe_2_ monolayer deposited on glass, 1p, and HMM substrates. **b,** The photoluminescence maps of WSe_2_ monolayer deposited on glass, 1p, and HMM substrates. Scale bar is 5 μm. For the PL map measurements, double-axis scanning galvo mirrors were used with a 4-f confocal alignment system. The PL emission was collected using a ×100 objective lens with the numerical aperture of 0.90, and sent to either an avalanche photodiode (Excelitas SPCM AQRH 13) or a momochromator/CCD (Princeton Instruments PIXIS 400 BRX). **c,** PL lifetime measurements of 2D WSe_2_ flake on top of HMM for different positions. HMM-reported is in the current main text. HMM-1 and HMM-2 are PL decays measured at two different positions within single 2D WSe_2_ flake.


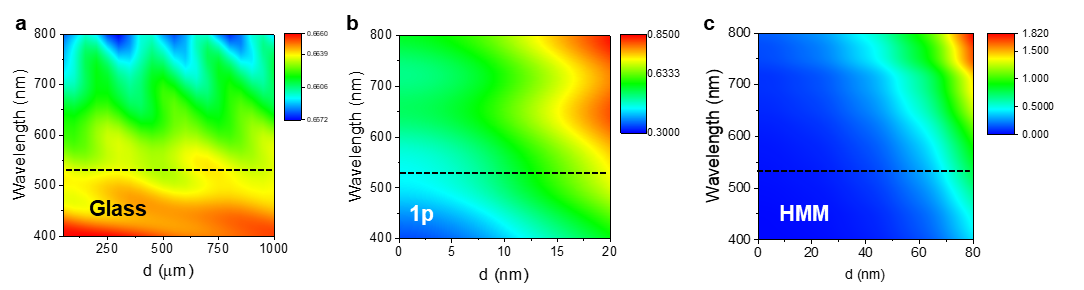


**Figure S6.** Numerical simulations of electric field intensity distributions along z-direction (normal to surface) for glass (a), 1p (b), and HMM (c) as a function of wavelength, calculated by FDTD simulations. Dashed line indicates excitation wavelength 532 nm. Because of extremely thin thickness of WSe_2_ monolayer (less than 1 nm), we could ignore the optical constant of WSe_2_ monolayer when calculating the field intensity.


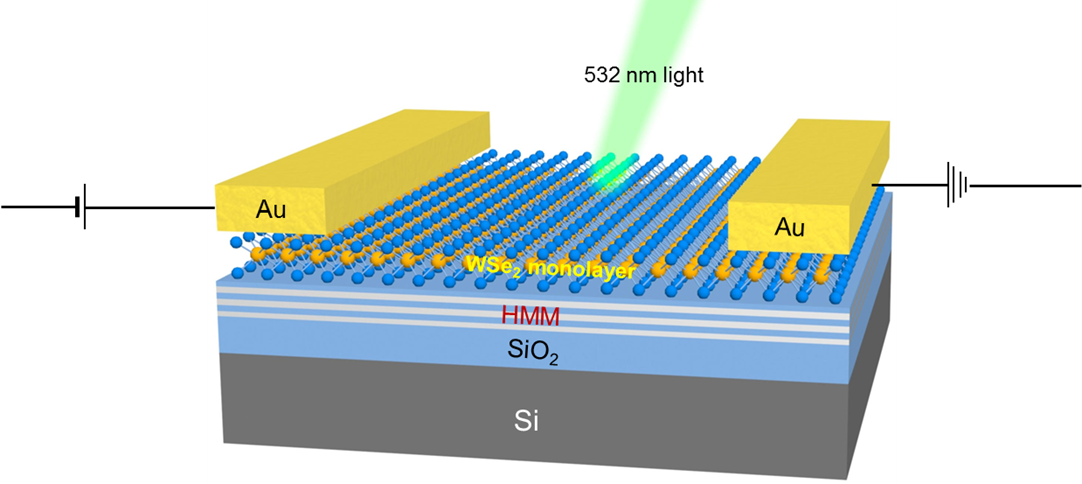


**Figure S7.** The schematic description of photodetector devices depending on the substrates where WSe_2_ monolayers are used as active materials. To measure the photocurrent, we applied a bias voltage to the samples using a source measurement unit (Keithley 2450) while they were illuminated at 532 nm.

**Figure S8.** Comparison of photocurrent measurements in the photodetector structure (shown in Figure S7) for old batch of HMM (reported, blue) and different batch of HMM (green).

**Table S1.** Summary of time constants obtained by bi-exponential fitting.

|  | $\tau_{1}$ ($ns$) | $\tau_{2}$ ($ns$) |
| --- | --- | --- |
| Glass | 0.27 (29 %) | 1.07 (71 %) |
| 1p | 0.33 (27 %) | 2.09 (73%) |
| HMM | 0.32 (27%) | 1.93 (73%) |

**Supplemental Note I: Discussion on the behavior of radiative and nonradiative decay rates**

Let us elaborate on that point by considering the equation for PLQY ($\Phi$).

$$\Phi=k_{r}\tau=\frac{k_{r}}{k_{r}+k_{nr}} (S1)$$

We can rewrite the above equation as follows

$$\Phi=\frac{1}{1+\beta} (S2)$$

where $\beta$ is $k_{nr}/k_{r}$. Based on plots shown in Figure 3b, we recognize that $\beta$ increases in the presence of metallic substrates, leading to a decrease in $\Phi$. Therefore, our experimental observation shows that the significant decrease in $k_{r}$ due to destructive image dipole interaction is responsible for the reduction of $\Phi$.

We now discuss about the quantitative estimation of the variation of $k_{r}$ and $k_{nr}$ using the relationship given in Eq. (R1). First, let us rewrite Eq. (R1) as,

$$\Phi=\frac{k_{r}}{k_{r}+k_{nr}}\to k_{nr}=k_{r}\left( \frac{1}{\Phi}-1 \right) (S3)$$

For glass substrate, we could estimate $k_{nr}$, substituting $\Phi=0.05$ into Eq. (R3), as

$$k_{nr}^{Glass}=k_{r}\left( \frac{1}{0.05}-1 \right)=19k_{r}.$$

Then, the values of $k_{nr}^{1p}$ and $k_{nr}^{HMM}$ could be estimated by substituting 59% and 61 % decreases of $k_{r}$ ($k_{r}\to0.4k_{r}$) with 18 % and 29 % decreases in $\Phi$ for 1p and HMM substrates, respectively, as

$$k_{nr}^{1p}={0.41k}_{r}\left( \frac{1}{0.04}-1 \right)={10.25k}_{r}$$

$$k_{nr}^{HMM}={0.39k}_{r}\left( \frac{1}{0.036}-1 \right)={10.43k}_{r}$$

Hence, the ratios of $k_{nr}$ on glass substrate to those on metallic substrates are

$$\frac{k_{nr}^{1p}}{k_{nr}^{Glass}}=\frac{10.25k_{r}}{19k_{r}}=0.54, \frac{k_{nr}^{HMM}}{k_{nr}^{Glass}}=\frac{10.43k_{r}}{19k_{r}}=0.55,$$

which show 46 % and 45 % decreases in $k_{nr}$ in the presence of 1p and HMM substrates, respectively.

**Supplemental Note II: Assigning the decay times obtained by fitting based on trap states**

In this section, we discuss the meaning of two distinct decay times shown in Table 1 by adopting the previous study reporting a quantitative description of charge-carrier dynamics in defective WSe_2_ monolayers based on a first-principles investigation [J. Am. Chem. Soc. **141**, 10451 (2019)]. According to this study, three of the most probable defects, namely, W vacancies, Se vacancies, and Se-W antisites are pronounced. It demonstrated that Se vacancies slow down recombination process by nearly an order of magnitude relative to defect-free samples by breaking the monolayer’s symmetry and thereby reducing the spectral intensity of the A_1g_ phonon mode. On the other hand, W vacancies and Se-W antisites accelerate recombination by introducing new phonon modes that strongly couple to electron and hole dynamics. As a result, four different recombination time scales, 16 ps, 150 ps, 400 ps, and 3.1 ns were reported. First two times correspond to trap-assisted recombination caused by W vacancies and Se-W antisites, respectively. 400 ps and 3.1 ns correspond to radiative recombination, and long-lived electrons in the trap states formed by Se vacancies, respectively.

Comparing these values with ours (Table S1), we can see that

1. $\tau_{1}$ likely corresponds to trap-assisted recombination from W vacancies and Se-W antisites combining with radiative recombination.
2. $\tau_{2}$ likely corresponds to radiative recombination combining with long-lived electrons in the trap states formed by Se vacancies.
